# Sterile α Motif Domain Containing 9 Is a Novel Cellular Interacting Partner to Low-Risk Type Human Papillomavirus E6 Proteins

**DOI:** 10.1371/journal.pone.0149859

**Published:** 2016-02-22

**Authors:** Jia Wang, Crystal Dupuis, Stephen K. Tyring, Michael P. Underbrink

**Affiliations:** 1 Department of Microbiology & Immunology, University of Texas Medical Branch, Galveston, Texas, United States of America; 2 Department of Otolaryngology, University of Texas Medical Branch, Galveston, Texas, United States of America; 3 Department of Dermatology, University of Texas Health Science Center, Houston, Texas, United States of America; International Centre for Genetic Engineering and Biotechnology, ITALY

## Abstract

Low-risk type human papillomavirus (HPV) 6 and 11 infection causes recurrent respiratory papillomatosis (RRP) and genital warts. RRP is the most common benign tumor of the larynx in children with frequent relapses. Repeated surgeries are often needed to improve vocal function and prevent life-threatening respiratory obstruction. Currently, there are no effective treatments available to completely eliminate these diseases, largely due to limited knowledge regarding their viral molecular pathogenesis. HPV E6 proteins contribute to cell immortalization by interacting with a variety of cellular proteins, which have been well studied for the high-risk type HPVs related to cancer progression. However, the functions of low-risk HPV E6 proteins are largely unknown. In this study, we report GST-pulldown coupled mass spectrometry analysis with low-risk HPV E6 proteins that identified sterile alpha motif domain containing 9 (SAMD9) as a novel interacting partner. We then confirmed the interaction between HPV-E6 and SAMD9 using co-immunoprecipitation, proximity ligation assay, and confocal immunofluorescence staining. The *SAMD9* gene is down-regulated in a variety of neoplasms and deleteriously mutated in normophosphatemic familial tumoral calcinosis. Interestingly, SAMD9 also has antiviral functions against poxvirus. Our study adds to the limited knowledge of the molecular properties of low-risk HPVs and describes new potential functions for the low-risk HPV E6 protein.

## Introduction

Human Papillomavirus (HPV) infections are responsible for malignant and benign tumors of mucosal and cutaneous squamous epithelia. Within the mucosal-tropic HPV group, certain HPV types (16,18) are categorized as high-risk types because of their ability to transform cells and cause cervical cancer, anogenital cancers and head and neck oropharyngeal cancers [1]. Low- risk mucosal-trophic HPV types, such as 6 & 11, do not possess the ability to transform cells. These particular HPV types are responsible for causing anogenital warts and recurrent respiratory papillomatosis (RRP), which cause proliferation of benign tumors in infected epithelium. RRP is commonly associated with significant proliferative growth and relentless recurrence of papillomas in vital laryngeal structures in both children and adults [2]. Currently, there is no curative treatment for these low-risk HPV infections, and management of disease is especially difficult in cases of RRP. The standard treatment for benign papilloma requires repeated surgical removal of the tumors, which imposes a significant economic burden to the healthcare system. It is estimated that the medical costs of these infections is approximately 0.5 billion dollars in United States annually [3].

The HPV oncoprotein E6 is a small protein of about 18 kDa, which forms two zinc finger-like structures that are conserved in both high-risk and low-risk HPV types. Structural analysis of the N and C-terminal halves of HPV-16 E6 suggests that the two zinc binding domains face each other and form a pseudodimer structure [4]. High-risk HPV E6 binds to LXXLL motif containing proteins, including the E6 associated protein (E6AP), through the hydrophobic pocket formed by the two zinc binding domains and the linker helix [5]. High-risk HPV E6s also contain an X-S/T-X-V/L motif at their c-terminus that binds to PDZ domain proteins [6–8], which is absent in low-risk HPV E6 proteins.

Despite the conserved structure of E6 in high and low-risk HPV types, significant differences in the ability to disrupt cellular function remain. The most well-known function of high-risk E6 proteins is to form a complex with the E3 ubiquitin ligase, E6AP, and the tumor suppressor, p53, to target p53 for poly-ubiquitination and proteasomal degradation [9, 10]. However, low-risk HPV E6 proteins do not cause the degradation of p53, despite an ability to bind both E6AP and p53 [11]. Additionally, high-risk HPV E6 protein can increase the efficiency of immortalization of human keratinocytes induced by E7 protein, while low-risk HPV E6 proteins cannot [12, 13]. A considerable amount of evidence has established multiple functions of high-risk type E6 proteins via their interaction with cellular proteins, such as up-regulating telomerase activity, inhibiting apoptosis, and disrupting cell polarity [14, 15]. These interactions are specific to the high-risk E6 proteins and have not been shown in low-risk HPV types.

To date, few interacting partners of low-risk E6s have been described, and most of these are also conserved in high-risk E6 proteins. Both low-risk and high-risk E6 proteins interact with E6AP [16, 17], and MCM7, a component of the replication licensing factors [18, 19]. HPV-6 and 18 E6 proteins interact with Gps2, a papillomavirus E2 dependent transcription coactivator [20]. HPV-11 and 16 E6 proteins interact with TRIP-Br1, a transcription factor, to stimulate the transactivation of E2F1/DP1 cell regulatory pathway [21], and p73 to inhibit its transactivation functions [22]. HPV-11 and high-risk E6 proteins destabilize TIP60, a histone acetyltransferase to abrogate p53-dependent apoptotic pathway activation [23], and Bak, an apoptotic Bcl-2 family member [24]. Zyxin is the only protein that has been described to bind solely to low-risk E6 proteins [25].

Considering the limited knowledge of low-risk E6 interacting proteins, we screened for cellular host proteins that interacted with low- risk HPV E6 proteins and identified a new binding partner of low-risk HPV E6 proteins, known as sterile α motif domain containing 9 (SAMD9). SAMD9 is a potential tumor suppressor, and is down-regulated in a variety of neoplasms [26]. The *SAMD9* gene is also deleteriously mutated in the hereditary disorder, normophosphatemic familial tumoral calcinosis (NFTC) [27], which is characterized by calcified tumors in skin and mucosa, painful ulcerative lesions and severe skin infections [28]. Our findings report a novel target of low-risk HPV E6 proteins, which adds to the limited studies on the low-risk HPV E6 proteins.

## Materials and Methods

### Cell culture

TERT-immortalized human epidermal keratinocytes N/TERT-1 cell line were obtained from Dr. James Rheinwald (Dept. of Dermatology, Harvard Skin Disease Research Center). This cell line was then used to generate stable HA tagged HPV-E6 expressing cell lines. N/TERT-1 cells and generated N/TERT-1 cells expressing HA tagged E6 cell lines were maintained in EpiLife medium (Life technologies) containing human keratinocyte growth supplement (HKGs) and penicillin-streptomycin (100U/ml). HEK293 (American Type Culture Collection) was also used to generate stable HA tagged HPV-E6 expressing cell lines. HEK293 cells and generated HEK293 cells expressing HA tagged E6 cell lines were maintained in Dulbecco’s modified Eagle’s medium (Gibco) containing 10% fetal bovine serum and penicillin-streptomycin.

### Plasmids

GST tagged E6 plasmids were generated by inserting E6 genes into pDEST15 vectors with the Gateway recombination system (Life technologies). HPV E6 genes were amplified by PCR using plasmids containing full-length HPV-6 (gift from Dr. Ethel-Michele de Villiers, German Cancer Research Center), -11 (gift from Dr. Louise Chow, University of Alabama at Birmingham), and -16 (American Type Culture Collection). All expressing constructs were confirmed by DNA sequencing. Plasmids MSCV-N-GFP, MSCV-N-6bE6, MSCV-N-11E6, and MSCV-N-16E6 were gifts from Dr. Karl Munger (Addgene plasmids #37855, 37915, 37872, 37875) [29] were used for HA tagged E6 stable cell line generation. pGEX 4T1 hBad was a gift from Dr. Harish Srinivas (Addgene plasmid #35567) [30].

### GST-E6 purification

pDEST15-E6 plasmids and pGEX 4T1 hBad were transformed into BL21-AI Escherichia coli competent cells (Life technologies). Single colonies were inoculated in LB medium containing 200ug/ml carbenicillin and grown overnight at 37°C. Then the cultures were expanded until the OD600 of the culture reached 0.4–0.6. Once obtained, GST-E6 proteins were induced by adding arabinose to reach the final concentration at 0.2%. GST-Bad protein was induced by 1mM IPTG. After 4 hours of induction, E. Coli cells were harvested, subjected to probe sonication at 50% power for 1.5 minutes and further lysed with 0.1% Triton X-100/PBS at 4°C for 30min. The supernatant was collected by centrifugation and incubated with pre-equilibrated glutathione sepharose 4B beads (GE) at 4°C for 1 hr with end over end rotation. The bead slurries were then washed four times with PBS with protease inhibitor tablets (Roche). GST-E6 proteins were eluted two times with 20mM glutathione (GSH), 50mM Tris-HCl, pH 8.0. A Zeba Spin Desalting Column (7K MWCO, Pierce) was then used to elute the purified GST-E6 proteins into dialysis buffer (5 mM Tris-HCl pH7.4, 100 mM KCl, 0.5 mM EDTA, 1 mM DTT, 5% glycerol, 0.1% NP40, and protease inhibitor tablets). The concentrations of purified GST-E6 proteins and GST-Bad were determined by DC protein assay (Bio-rad).

### GST pulldown assay

Equal amounts of GST-E6 proteins and GST-Bad in dialysis buffer were incubated with N/TERT-1 cell lysates pre-cleared with sepharose 4B beads at 4°C for 1hr with end over end rotation. Then pre-equilibrated glutathione sepharose 4B beads were then added into the protein-lysate mixture and incubated at 4°C for 2hr. Beads were pelleted by centrifugation and washed three times with binding buffer (1x PBS, 1% NP40, 2mM DTT, and protease inhibitor tablets). After final wash, 4x LDS sample buffer (Life technologies) was added, boiled for 5 minutes, and supernatant were collected and stored at -80°C until further use.

### Mass spectrometry

The supernatant from the GST pulldown assays were separated on NuPAGE 4–12% Bis-Tris gels (Life technologies) and stained with SimplyBlue SafeStain (Life technologies). Whole lane of protein bands were cut into 1cm pieces in length and sent to UTMB Mass Spectrometry Core for LC-MS/MS analysis. Three independent runs for GST-6E6 and GST-11E6, two independent runs for GST-16E6 and GST-bad were performed for LC-MS/MS analysis.

### Protein functional analysis

Proteins identified by LC-MS/MS with average unique peptide number equal or more than 4 were analyzed using Ingenuity Pathway Analysis (IPA; Qiagen) by UTMB bioinformatics program. Functional annotation of the proteins and the network were constructed using IPA.

### Retrovirus production and infection

Retroviruses were produced by transfecting MSCV-N-E6 plasmids and pVSV-G into Gp2-293 packaging cell line (Retro-X universal Expression System, Clontech). The supernatant was collected and viruses were concentrated using ultracentrifugation. N/TERT-1 cells were infected at around 50% confluence with the addition of Polybrene for overnight. The medium was replaced next day. Puromycin (0.5ug/ml) selection started at day 2 post infection and lasted for 2 weeks. The selected N/Tert-1 expressing HA tagged HPV-E6 cell lines were propagated for further studies.

### Transient and stable transfection

HEK293 cells (ATCC) were transfected with MSCV-N-GFP, MSCV-N-6E6, MSCV-N-11E6, and MSCV-N-16E6 plasmids using Fugene 6 (Promega). Forty-eight hours post transfection, cells were lysed and used for co-immunoprecipitation assay. For stable transfection, HEK293 cells were transfected with the same amount of MSCV-N-GFP and MSCV-N-E6 constructs using Fugene 6 (Promega) and selected with puromycin for 2 weeks. Then single clones were then isolated and propagated for co-immunoprecipitation assays.

### Co-immunoprecipitation (Co-IP)

HEK293 cells that transiently or stably express HA-tagged E6 proteins were harvested and lysed in 0.5% NP40 lysis buffer. Pre-equilibrated anti-HA conjugated agarose beads (A2095, Sigma) were then incubated with the cell lysates containing the same amount of proteins for 4 hours with end over end rotation at 4°C. The beads were washed four times with NP40 lysis buffer and boiled in 2x SDS sample buffer (Sigma) for 5 min. These samples were then separated on NuPAGE 4–12% Bis-Tris gel and immuno-blotted with following antibodies: anti-HA (Cell Signaling) and anti-SAMD9 (Sigma).

### Proximity Ligation Assay

N/TERT-1 cell line and N/TERT-1 cells expressing MSCV-N-6E6, MSCV-N-11E6, and MSCV-N-16E6 were grown on Lab-Tek Chamber slide for 24 h. Cells were then fixed using 4% PFA and permeabilized with 0.5% Triton X-100/PBS. Duolink In Situ Red Starter Kit Mouse/Rabbit (Sigma) was used for the proximity ligation assay. Rabbit anti-SAMD9 (Sigma), mouse anti-HA monoclonal (Sigma) antibody pairs and rabbit anti-HA monoclonal (cell signaling), mouse anti-E6AP monoclonal (Sigma) antibody pairs were used as primary antibodies. Images were obtained using Olympus FV1000 confocal microscope with 60 x oil lens. The excitation and emission wavelengths of PLA signals were 594 and 624 nm. Cell nuclei were stained with 4′,6-diamidino-2-phenylindole (DAPI). The PLA signals were counted using image J, and a total of 80 to 120 cells were examined.

### Immunofluorescence Staining

N/TERT-1 and N/TERT-1 cells expressing MSCV-N-11E6 were grown on Lab-Tek Chamber slide for a day. Cells were fixed using 4% PFA and permeabilized using 0.5% Triton X-100. After blocking, cells were incubated with diluted mouse anti-HA (sigma) and rabbit anti-SAMD9 (sigma) at 4°C overnight. After three washes, cells were incubated with Alexa Fluor 568 labeled goat anti-mouse secondary antibody (ThermoFisher Scientific) and Alexa Fluor 488 labeled goat anti-rabbit secondary antibody (ThermoFisher Scientific) for 1 hr in room temperature. Prolong gold antifade mountant with DAPI (ThermoFisher Scientific) was used for mounting. Confocal images were acquired using a Zeiss LSM510 confocal microscopy with a 63x water lens. Excitation wavelengths were 364nm for DAPI, 488nm for Alexa Fluor 488, 543nm for Alexa Fluor 568 respectively.

## Results

### Identification of cellular proteins that interact with low-risk HPV E6 proteins

Previously, cellular interacting proteins of low-risk HPV E6 proteins were either identified by yeast two-hybrid screen [19–21] or by parallel studies with high-risk HPV E6 functions driven by hypothesis [16, 22–24]. Although several systematic studies using affinity purification (AP) coupled with liquid chromatography tandem mass spectrometry (LC-MS/MS) have identified novel cellular proteins that interact with other HPV types, few were found to bind to low-risk E6 proteins [29, 31]. To investigate the low-risk HPV E6 interacting proteins, we used GST-pulldown coupled LC-MS/MS [32] to screen for potential candidates. The E6 proteins from two low-risk types, 6 and 11, and one high-risk type (16) were fused with GST at the N-terminus and then used for GST-pulldowns. GST tagged Bcl-2 associated death promoter (Bad), a member of Bcl-2 family was used as a negative control because of its similarity in size to the HPV E6 protein. Each GST-pulldown was performed and analyzed at least twice. The Coomassie Blue stained binding patterns from the individual GST-pulldown assays are shown in S1 Fig.

**Fig 1:**
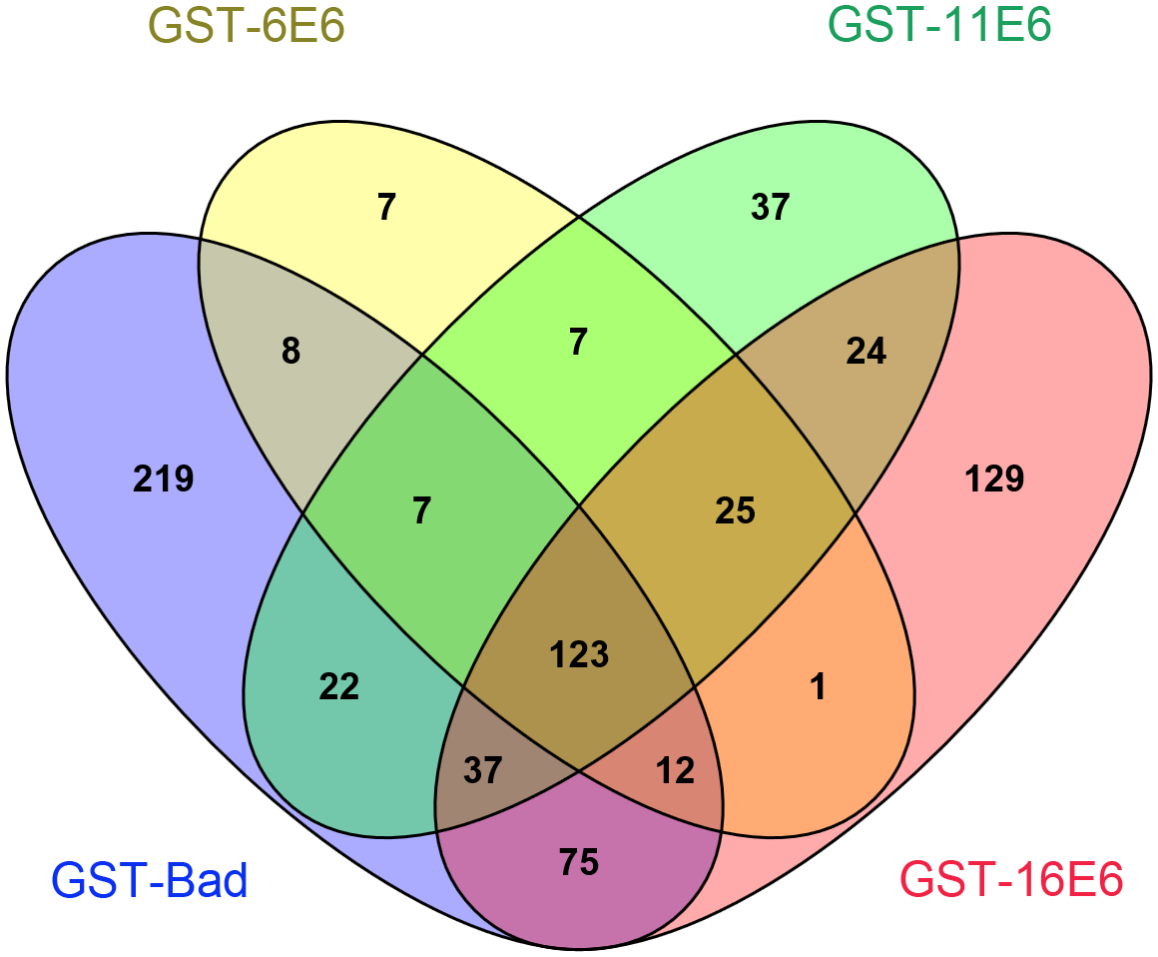
Venn analysis of proteins in complex with GST-E6s. The numbers of cellular proteins detected in complex with GST-6E6 were shown in venn analysis.

Venn analysis of the proteins detected by LC-MS/MS with the different GST fused bait proteins show that 503 cellular proteins potentially interact with GST-Bad. Among these, 284 proteins intersect both GST-Bad and GST-E6 proteins and were therefore subtracted from GST-E6 protein binding datasets. After subtraction, there were 40 cellular proteins detected in complex with GST-6E6, 93 proteins with GST-11E6, and 179 proteins with GST-16E6. Within this dataset, seven proteins were exclusively detected in complex with GST-6E6, 37 proteins with GST-11E6, and 129 proteins with GST-16E6, using the average unique peptide number equal to 4 as the cutoff (Fig 1). Known HPV-16E6 interacting proteins, including p53 [17], E6AP [10], MAGI1 [33], SCRIB [7], PTPN3 [8] and DLG1 [6] were also present in the GST-16E6 protein complex obtained in our dataset. The full list of proteins detected in complex with each of the GST-E6 proteins is shown in S1 Table.

**Fig 2:**
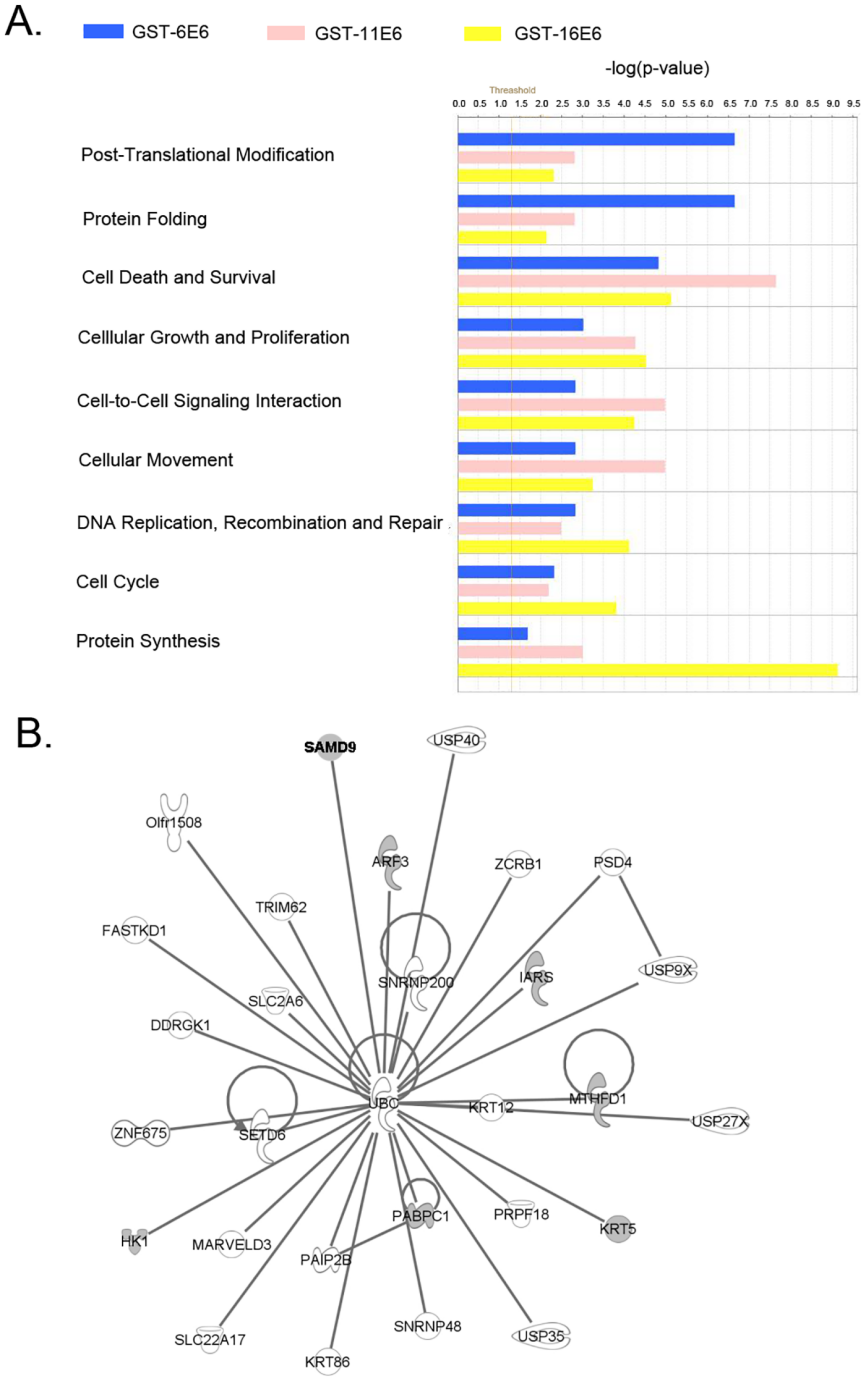
IPA analysis of host proteins in complex with GST-HPV E6 proteins. (A) Biological functions of the identified cellular proteins by IPA. The significance level was calculated by using Fisher’s exact test. The Threshold line represents *p* < 0.05. (B) A network generated by IPA. Grey nodes showed proteins found in complex with GST-11E6. SAMD9 is shown in bold.

To further delineate potential biological functions and determine theoretical networks associated with candidate E6 interacting proteins, we used ingenuity pathway analysis (IPA) to analyze the functions of proteins that were identified in complex with E6 proteins from each HPV type. Consistent with previous studies [34, 35], protein synthesis (p = 7.4x10^-10^–7.19x10^-3^ with 23 focus molecules), and cell death and survival (p = 7.43x10^-6^–1.43x10^-2^ with 57 focus molecules) were found to be the top functions affected by HPV-16 E6. Cell death and survival (p = 2.25x10^-8^–2.57x10^-2^ with 37 focus molecules) was the top function of proteins that complexed with GST-11 E6. In contrast, post-translational modification (p = 2.22x10^-7^–2.05x10^-2^ with 6 focus molecules), and protein folding (p = 2.22x10^-7^–2.33x10^-6^ with 5 focus molecules) were the top functions of cellular proteins found in complex with GST-6E6 (Fig 2A) found in our study. Based on published literature and established biological networks, IPA is able to fit the candidate proteins that complex with GST-HPV E6 proteins into various cellular networks. From this analysis, we determined that the cell death and survival and developmental disorder network was one of the top networks identified from our datasets. Therefore, we overlaid the proteins identified in the GST-11 E6 complex with this network and found that several potential HPV-11 E6 protein interacting proteins were grouped with ubiquitin C (Fig 2B). Among them, the sterile α motif domain-containing 9 (SAMD9) protein, which has been shown to affect cell proliferation and apoptosis [26], was chosen for further study.

### Low-risk HPV E6 proteins interact with SAMD9

We used co-immunoprecipitation (Co-IP) analysis to confirm this interaction in vivo. HEK 293 cells were transiently transfected with N-terminal HA-tagged E6 proteins from HPV-6, -11 and -16 or GFP protein as control. These cells were then lysed and HA-E6 proteins or HA-GFP were captured on anti-HA agarose beads. The samples were run on SDS-PAGE, followed by transfer onto PVDF membrane and blotted against anti-HA and anti-SAMD9 antibodies.

**Fig 3:**
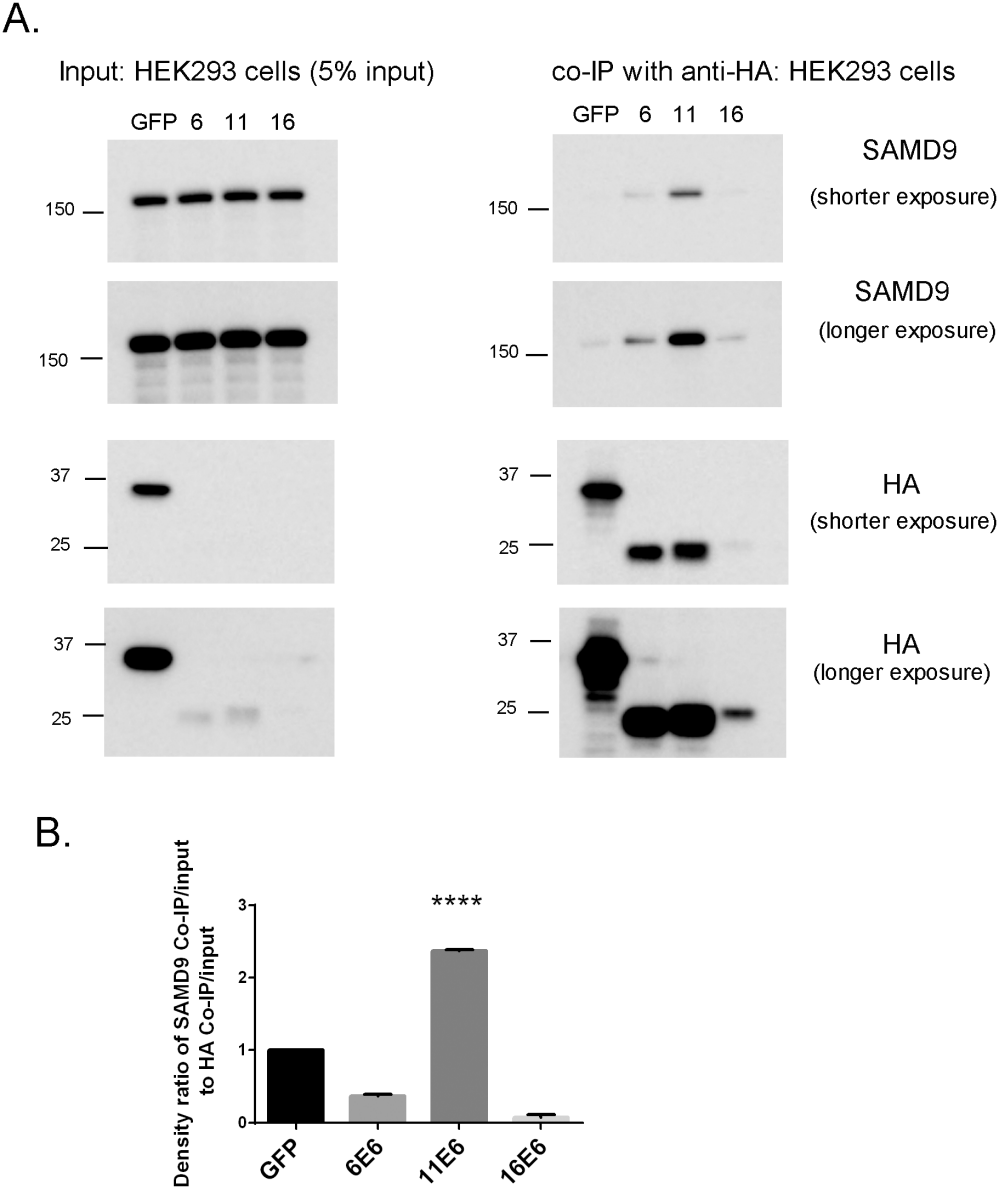
HPV-11 E6 protein interacts with SAMD9 in HEK293 cells. (A) Input and co-immunoprecipitation of HA tagged E6 proteins in HEK293 cells that transiently express HA tagged GFP, 6E6, 11E6 and 16E6 proteins. Western blots were used to detect SAMD9 and HA in whole cell lysates and co-immunoprecipitated complexes using these cell lines. (B) The association between SAMD9 and E6 proteins was quantified and the density ratio of co-immunoprecipitated SAMD9 normalized by Immunoprecipitated HA was calculated. **** represents *p*<0.0001 when compared to control group. Student’s t-test was used for statistical analysis.

We found that the expression levels of HA-6E6 and HA-11E6 were comparable and were much higher than HA-16E6. This is in agreement with previous reports demonstrating that 16E6 possessed the lowest steady-state level when compared to 6E6 and 11E6 under transient transfection conditions [36]. All HA-E6 expression levels were significantly lower than HA-GFP control expression. The expression levels of SAMD9 were similar in whole cell lysates from each of the E6 cell lines and GFP control cells. After Co-IP, SAMD9 had the strongest signal in cells expressing HPV-11 E6, a weaker signal in cells expressing HPV-6 E6, and minimal signals in cells expressing either HPV-16 E6 or GFP (Fig 3A). Quantification of co-immunoprecipitated SAMD9 normalized by immunoprecipitated HA showed a significant increase in the association between SAMD9 and HPV-11 E6 compared to GFP control (*p*<0.0001)(Fig 3B). We also generated and isolated single clones of HEK 293 cells that stably expressed HA tagged HPV-E6 proteins and HA-GFP. Co-IPs using the highest expressing cell clones from each of the cell lines yielded similar results (S2 Fig). Taken together, our results indicate that low-risk type 11 preferentially interacts with SAMD9 protein.

**Fig 4:**
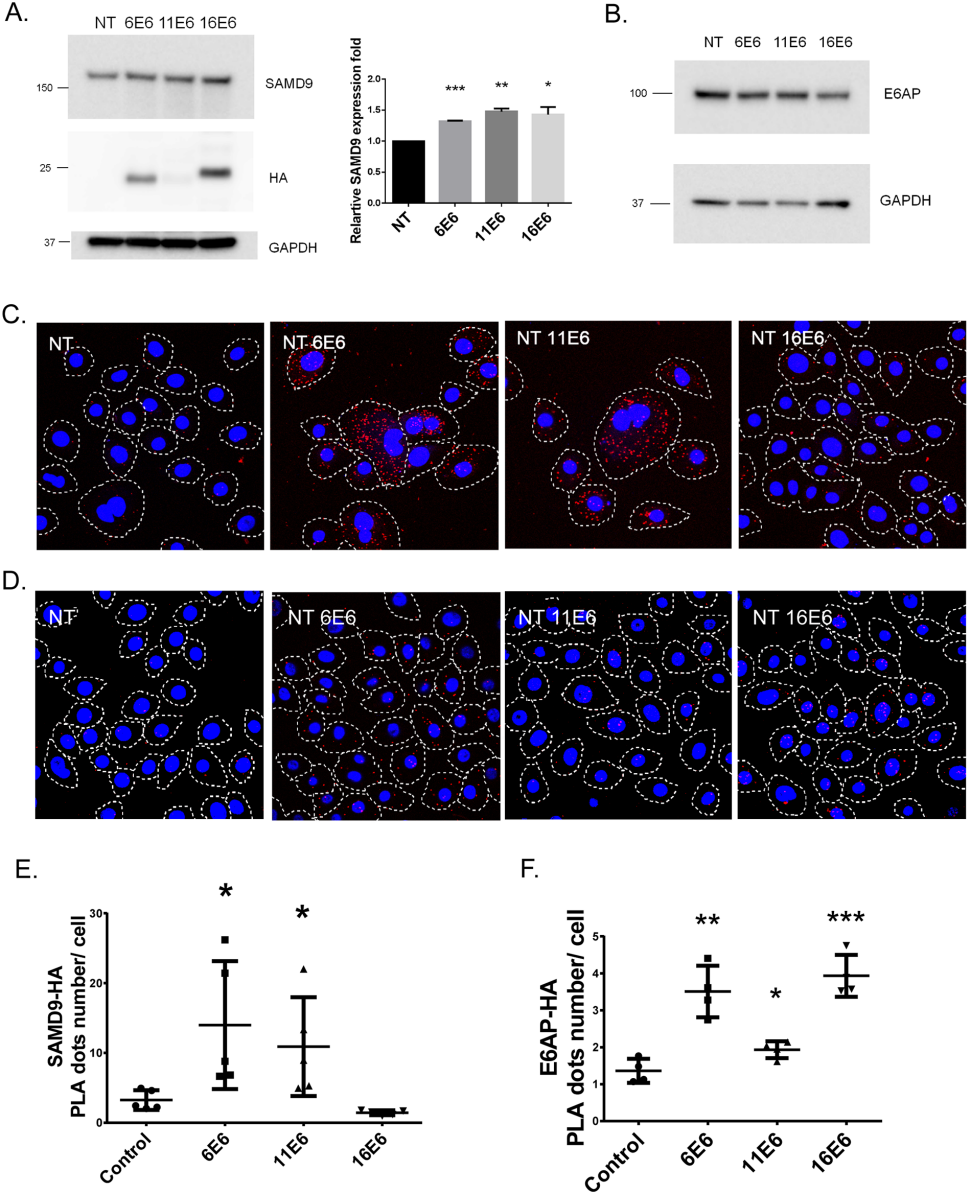
Low-risk HPV- E6 proteins interact with endogenous SAMD9 in human keratinocytes. (A) HA-E6 expression levels in N/Tert-1 and N/Tert-1 HA-E6 cells. Whole cell lysates were used for western blot to detect the HA, SAMD9 and GAPDH. Quantification of SAMD9 expression level in control and HA-E6 expressing cells. (B) E6AP expression levels in N/Tert-1 and N/Tert-1 HA-E6 cells. (C) PLA assays were performed in N/Tert-1 cells, and N/Tert-1 cells expressing HA tagged HPV-6 E6, HPV-11 E6 and HPV-16 E6. Anti-HA and anti-SAMD9 were used as primary antibodies. Red fluorescence dot represents single interaction between these two proteins. Nuclei were stained with DAPI in blue. The images were acquired on an Olympus FV1000 confocal microscope by using a 60 x oil lens. (D) PLA assays using anti-HA and anti-E6AP pair were performed in these cell lines. Red fluorescence dot represents single interaction between these two proteins. Nuclei were stained with DAPI in blue. The images were acquired on an Olympus FV1000 confocal microscope by using a 60 x oil lens. (E) Quantification of the PLA signal of SAMD9-HA E6 interaction. Five different fields were used for the analysis and a total of 80 to 120 cells were examined. (F) Quantification of the PLA signal of E6AP-HA E6 interaction. A total of 80 to 120 cells were examined. * represents *p*<0.05, ** represents *p*<0.01 and *** represents *p*<0.001 when compared to control group. Student’s t-test was used for statistical analysis.

In order to validate the Co-IP results and also determine the cellular location of this interaction, we used the proximity ligation assay (PLA) to investigate SAMD9 –E6 interaction in N/Tert-1 keratinocytes. The expression levels of the HA-tagged E6 proteins and endogenous SAMD9 in the N/Tert-1 keratinocytes are shown in Fig 4A. There is approximately a 1.5-fold increase of SAMD9 expression in HPV- E6 expressing cells when compared with normal keratinocyte control cells. A significant number of interactions between SAMD9 and HA-E6 were found in the low-risk E6 cells, compared to high-risk HPV-16 E6 expressing and control cells. Interestingly, the significant number of SAMD9 and HA-11E6 interactions was maintained despite a much lower level of HA-11E6 expression than the HA-6E6 and -16E6 cell lines used for this assay. Additionally, most of the interactions take place in the cytoplasm of the N/Tert-1 E6 expressing keratinocytes (Fig 4C). Quantitative analysis of PLA signals illustrates a comparable average PLA signal number per cell in HPV-6 and -11 E6 cells, which is significantly higher than that in HPV-16 E6 and control N/Tert-1 cells (p<0.05) (Fig 4E). As an additional positive control, we also used the PLA to investigate E6AP-E6 interaction in our HA-E6 expressing keratinocytes, since E6AP is known to bind both low (6, 11) and high (16) risk E6 proteins [16, 37]. We found a significant number of interactions between E6AP and HA-E6 in both high-risk and low-risk E6 cells, compared to control cells. Especially in HA-16E6 cells, the majority of the interactions take place in the nuclei (Fig 4D). When compared to control cells, HPV-16 E6 expressing cells have the highest interaction level (p<0.001), followed by HPV-6 E6 (p<0.01) and HPV-11 E6 (p<0.05) (Fig 4F), which seems to correlate with the level of HA-E6 expression in each cell line.

**Fig 5:**
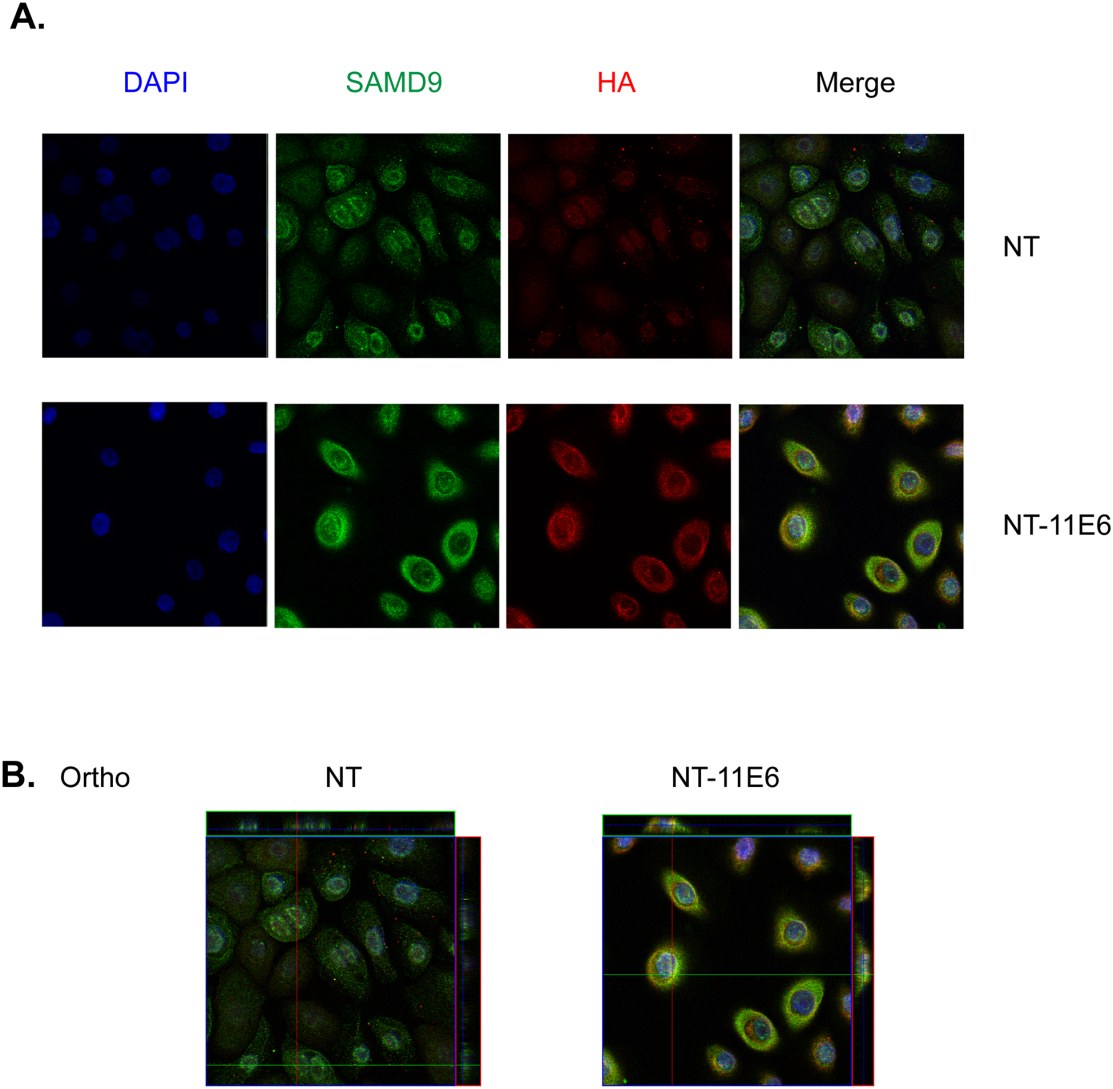
HPV-11 E6 colocalizes with SAMD9 in immortalized human keratinocytes. (A) N/Tert-1 cells and N/Tert-1 cells expressing HA tagged HPV-11 E6 protein were immunostained with anti-SAMD9 (green), anti-HA (red) and DAPI (blue). Cells were imaged by confocal microscopy with a 63x water lens. (B) A representative z-stack of images was collected from each cell lines stained with SAMD9 and HA-11 E6. An orthogonal section across the z-stack of images shows the co-localization of 11 E6 and SAMD9 (yellow area).

To determine whether HPV-11 E6 co-localizes with SAMD9 in N/Tert-1 cells or whether it alters SAMD9 localization, we performed immunofluorescence staining in N/Tert-1 control cells and N/Tert-1 cells stably expressing HA-tagged HPV 11 E6. As shown in Fig 5, 11 E6 proteins display a strong, primarily cytoplasmic staining pattern and a weak spotted intra-nuclear localization. We also observe punctate staining pattern of SAMD9 in cytoplasm and nucleus in both control and 11 E6 expressing cells. HPV 11 E6 did not significantly alter the localization of SAMD9. Confocal Z-stack analysis revealed that HA -11 E6 co-localizes with SAMD9 in the cytoplasm of the cell, which corroborates our previous PLA assay findings.

## Discussion

Previous researchers have often suggested that there were shared interacting partners between low-risk and high-risk E6 proteins [18, 20–24]. Zyxin is the only protein to our knowledge that has been reported to interact solely with low-risk HPV E6 protein (HPV-6) [25]. Based on the resolved structure of HPV-16 E6, surface property analysis of low-risk E6 proteins showed different physicochemical characteristics [4]. Therefore, we hypothesize that low-risk HPV E6 proteins may have different interaction partners in comparison to their high-risk HPVs counterparts. Here we report the identification of a novel protein, SAMD9 that interacts preferentially with low-risk E6 proteins. SAMD9 was initially identified using GST-pulldown and LC-MS/MS analysis and then validated with additional assays. Co-IP experiments show that the interaction between the E6 proteins and SAMD9 is only observed in low-risk E6 expressing cells. Similarly in human keratinocytes, despite reduced HPV-11 E6 expression levels compared to HPV-6 and HPV-16 E6, signal generated by the proximity ligation assays (PLA) is only observed in the low-risk HPV 6E6 and 11E6 expressing cells. Localization of PLA signal to the cytoplasm is consistent with previous studies that have individually shown that low-risk HPV E6 [38] and SAMD9 [26] both localize to the cytoplasm. Our studies reveal that the low-risk HPV E6 proteins preferentially interact with cellular SAMD9 protein within the cytoplasm of keratinocytes.

As low-risk HPV E6 proteins also bind to the LXXLL motif in E6AP [16], we examined whether the SAMD9 protein contains a similar LXXLL motif. In addition to a sterile alpha motif (SAM) domain near N-terminal region [26], there are three LXXLL sequences found in SAMD9. However, unlike the LXXLL motifs in E6AP, the amino acids in the XX positions of SAMD9 are not negative residues. In order to better understand the SAMD9 –E6 interaction, further study including mutagenesis of both the SAMD9 and low-risk HPV E6 proteins is needed to map the regions required for binding.

There are many interesting possibilities for study concerning the physiological significance of the interaction of low risk HPV E6 with SAMD9. Previous studies have shown that low-risk E6 protein is indispensable for maintaining episomal replication for HPV-11 [39], although an exact mechanism is currently unknown. Interestingly, SAMD9 has also been shown to be the antagonist of the myxoma virus host range factor M062R [40], as well as C7L and K1L proteins of vaccinia virus [41], which controls productive viral replication in human cells. SAMD9 has further been shown to associate with stress granules induced by infection with host range deletion mutants [42]. Although HPV is very different from poxvirus, it is still possible that SAMD9 may also participate in controlling low-risk human papillomavirus replication. We hypothesize that the E6 proteins from low-risk HPV types possibly antagonize the normal antiviral effects of SAMD9. Further studies will be needed to verify the physiologic role of SAMD9 –E6 interaction in low-risk HPV pathogenesis.

Besides anti-viral replication function, SAMD9 was implicated to control cancer cell death induced by inactivated Sendai virus particle (HVJ-E) or interferon (IFN) treatment [43], although the mechanism remains unclear. IFN-α has been used with success as an adjuvant therapy for RRP. However, there is controversial data on the effectiveness of IFN-α in long-term treatment of RRP [44–46]. It is certainly plausible that SAMD9 is an important molecular target, which may mediate the responsiveness of papilloma growth to IFN treatment.

Proteomic approaches have been widely used to study the interactions between HPV viral proteins and host [47]. Here we used the GST pulldown coupled LC-MS/MS to initially screen for novel binding partners that bind to low-risk HPV E6 proteins. Our low-risk E6 interacting proteins dataset were largely non-overlapping, when compared to those identified by tandem AP-MS or enzyme complementation assay in mammalian cells, due to the difference of methods used [29, 31, 48]. One of the limitations of the method employed here was the possibility of detecting non-specific interacting proteins, owing to exposing high levels of E6 proteins to all cellular proteins. However, we do expect the detection of cellular proteins that genuinely interact with low-risk HPV E6 proteins, as several known HPV-16 interacting protein were also identified by our method. Therefore, the initial screen provided us many more potential interacting proteins to both low-risk and high-risk E6 proteins, and further validation is certainly warranted for the vast majority of these.

In conclusion, we have identified a novel host protein SAMD9 that interacts with low-risk HPV 6 and 11E6 proteins in the cytoplasm of keratinocytes. Additional studies are currently underway to elucidate the physiological functions of the SAMD9 –E6 interaction. Given the importance of cellular machinery in HPV pathogenesis, counteracting critical interactions between E6 protein and cellular proteins, may offer novel therapeutic targets for low-risk HPV associated diseases.

## Supporting Information

S1 Fig Coomassie blue staining of GST-E6 and GST-bad pulldown elutions and inputs. (TIF)

S2 Fig Low-risk HPV-E6 proteins interact with SAMD9 in stable HEK293 cells. (A) Western blots of whole cell lysate of HEK293 cells stably expressing HA tagged GFP (control), 6E6, 11E6 and 16E6 proteins. (B) Co-immunoprecipitation using anti-HA agarose beads in these cell lines. Precipitated proteins were subjected to western blot to detect SAMD9, and HA.
(TIF)

S1 Table List of proteins in complex with GST-E6 proteins. (XLS)
