# Supplementary figures and images for: Sterile α Motif Domain Containing 9 Is a Novel Cellular Interacting Partner to Low-Risk Type Human Papillomavirus E6 Proteins
Source: PLoS One. 2016 Feb 22;11(2):e0149859. doi: 10.1371/journal.pone.0149859 (PMC4764768; doi:10.1371/journal.pone.0149859)

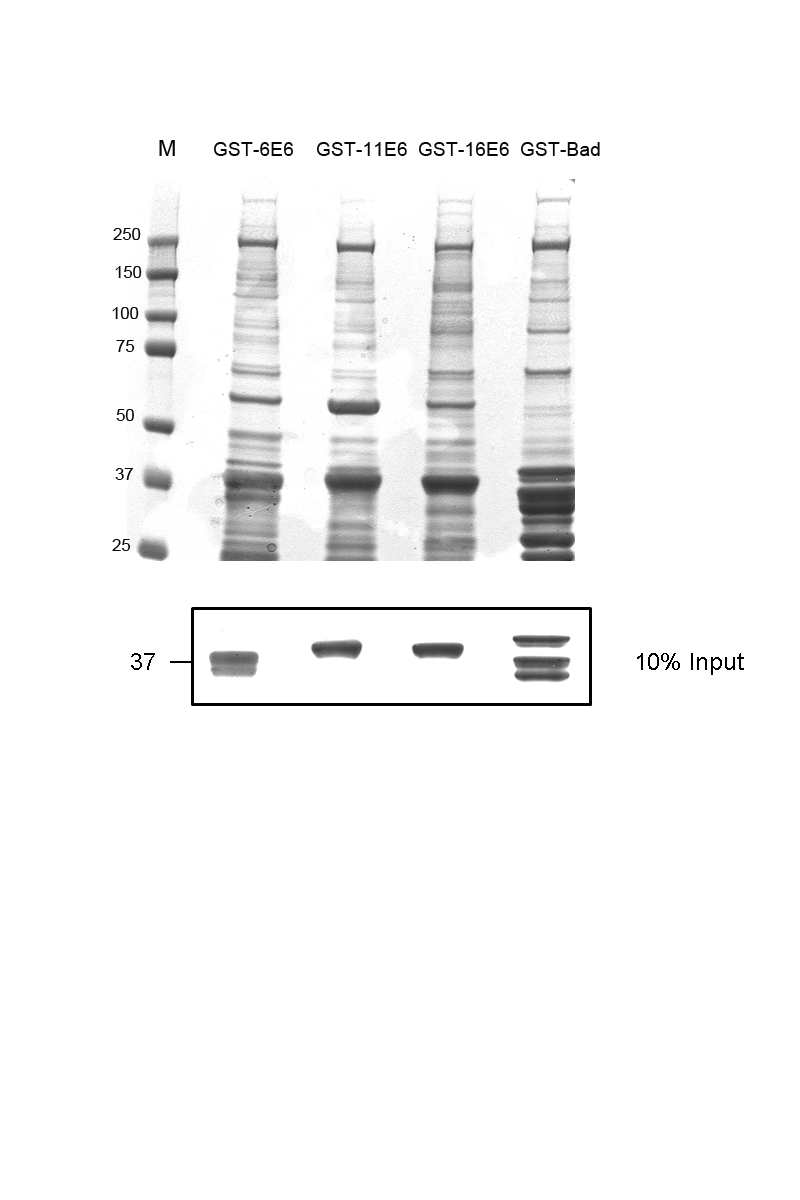

Supplement: S1 Fig — (TIF) [file pone.0149859.s001.tif]

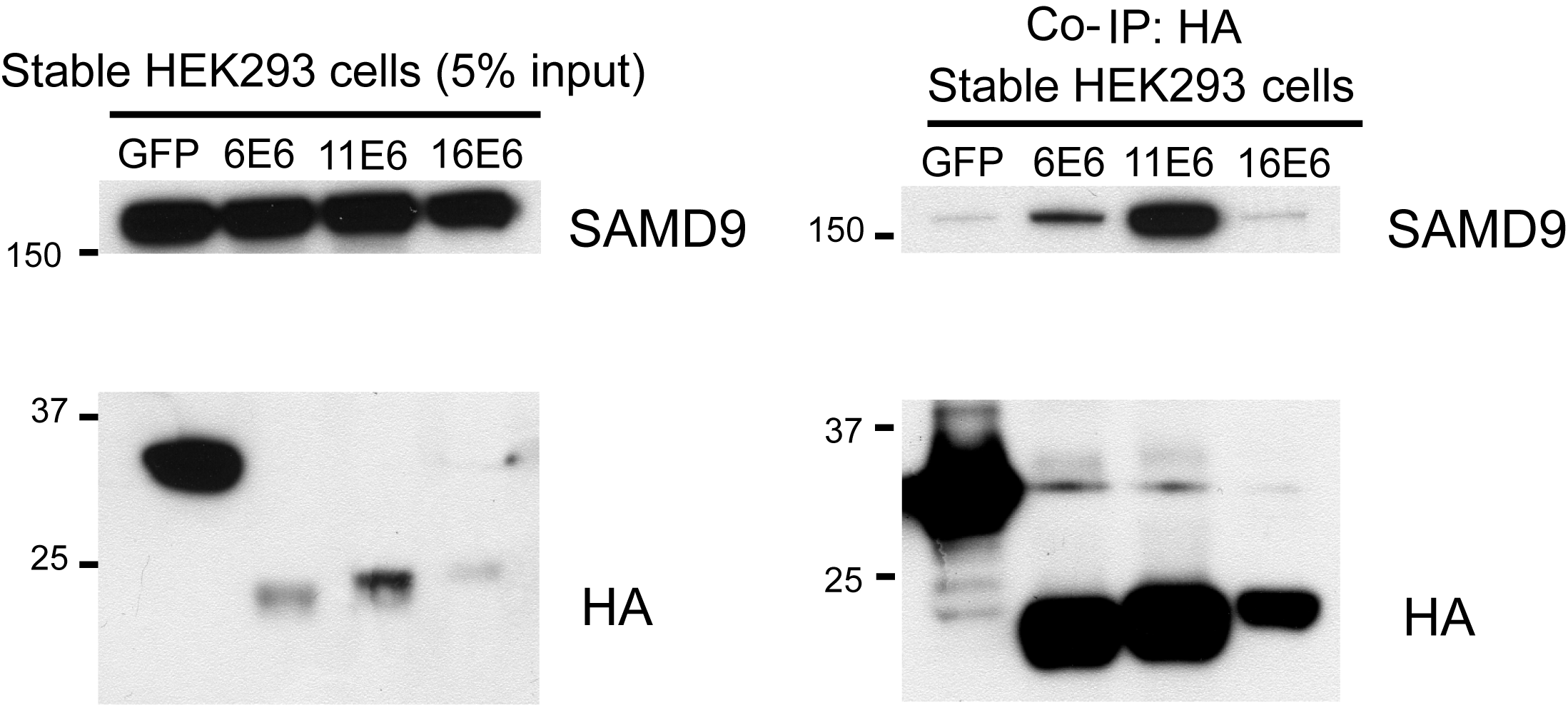

Supplement: S2 Fig — (A) Western blots of whole cell lysate of HEK293 cells stably expressing HA tagged GFP (control), 6E6, 11E6 and 16E6 proteins. (B) Co-immunoprecipitation using anti-HA agarose beads in these cell lines. Precipitated proteins were subjected to western blot to detect SAMD9, and HA. (TIF) [file pone.0149859.s002.tif]
